# Effects of step lengths on biomechanical characteristics of lower extremity during split squat movement

**DOI:** 10.3389/fbioe.2023.1277493

**Published:** 2023-11-10

**Authors:** Qingquan Song, Mujia Ma, Hui Liu, Xiaobin Wei, Xiaoping Chen

**Affiliations:** ^1^ School of Strength and Conditioning Training, Beijing Sport University, Beijing, China; ^2^ Biomechanics Laboratory, School of Sport Science, Beijing Sport University, Beijing, China; ^3^ China Institute of Sport and Health Science, Beijing Sport University, Beijing, China; ^4^ Centre for Sports Research, China Institute of Sport Science science, Beijing, China

**Keywords:** biomechanics, EMG, lower extremity, split squat, rehabilitation

## Abstract

**Objective:** To quantify the effects of increasing the step length of the split squat on changes in kinematics, kinetics, and muscle activation of the lower extremity.

**Methods:** Twenty male college students participated in the test (age: 23.9 ± 3.7, height: 175.1 ± 4.9). Data on kinematics, kinetics, and EMG were collected during split squat exercise at four different step lengths in a non-systematic manner. One-way repeated measurements ANOVA were used to compare characteristic variables of peak angle, moment, and RMS among the four step length conditions.

**Results:** The step length significantly changes the peak angles of the hip (*p* = 0.011), knee (*p* = 0.001), ankle (*p* < 0.001) joint, and the peak extension moment of the hip (*p* < 0.001), knee (*p* = 0.002) joint, but does not affect the ankle peak extension moment (*p* = 0.357) during a split squat. Moreover, a significant difference was observed in the EMG of gluteus maximus (*p* < 0.001), vastus medialis (*p* = 0.013), vastus lateralis (*p* = 0.020), biceps femoris (*p* = 0.003), Semitendinosus (*p* < 0.001), medialis gastrocnemius (*p* = 0.035) and lateralis gastrocnemius (*p* = 0.005) during four step lengths, but no difference in rectus femoris (*p* = 0.16).

**Conclusion:** Increases in step length of split squat had a greater activation on the hip extensor muscles while having a limited impact on the knee extensor muscles. The ROM, joint moment, and muscle activation of the lead limb in the split squat all should be considered in cases of individual preventative or rehabilitative prescription of the exercise. Moreover, the optimal step length for strength training in healthy adults appears to be more suitable when it is equal to the length of the individual lower extremity.

## 1 Introduction

The optimal exercise selection for improving, maintaining, and enhancing functional capacities involves aligning the demands of an exercise with the specific needs of the client or patient (Flanagan et al., 2003; Wu et al., 2020). Using an exercise for training or evaluation requires a full understanding of the mechanical demands imposed on the musculoskeletal system. Unfortunately, for many commonly used exercises, such as the split squat or lunge and their variations, the specific biomechanical characteristics are largely unknown, making exercise selection and progression decisions to be largely based on intuition and clinical experience.

A split squat, or a forward lunge, is a multijointed exercise of a closed kinetic chain used to improve the function or strength of the lower extremities (Graham, 2011; Severin et al., 2017; Williams et al., 2021). The main difference between a split squat and a forward lunge is foot movement and stability. In a split squat, both feet remain in a fixed position and the movement is mainly up and down, which can help increase the strength of the lower extremity in a relatively stabilized position. In contrast, a forward lunge requires one foot to move forward and then backward with each repetition, which requires more muscles for maintaining balance and stability, including the thigh, glutes, and calf muscles (Chapman, 2018). Although forward lunge have been shown to improve hamstring strength and sprint performance (Jönhagen et al., 2009). However, Split squats are frequently utilized towards the end of rehabilitation settings, especially following cruciate ligament reconstruction, in order to enhance the strength of the muscles in the lower extremities (Escamilla et al., 2008; Wu et al., 2021; Gokeler et al., 2022). In addition the static foot positioning of a split squat means that they can handle the load better and more safely than a forward lunge which requires moving a foot. This makes split squat a preferable exercise for beginners or patients with balance or coordination challenges.

According to a general statistic, joint injuries are very common in the athletic population, with an incidence of 10–35.5 injuries per 1,000 (Vannini et al., 2016). Most of the injuries were due to incorrect exercise execution, or overload (Costa et al., 2016), despite several available guidelines that describe the correct execution. Thus, it is important to understand how the mechanical demands are distributed across the joints and muscles when evaluating the appropriateness of the split squat exercise. Split squat guidelines typically include a focus on weight distribution between legs, step length, and the tibia angle. For a step length of the split squat, a common guideline in practice is to keep the front knee directly above the front foot (Haff and Triplett, 2016). However, these guidelines are based on the experience of a trainer rather than science-based evidence.

Recently, it was shown that the front tibia angle influences joint angles and loading conditions during the split squat exercise (Schütz et al., 2014). Suggest that the tibia angle of 60° should be chosen for high loading of the front leg. As in real practice, it is difficult to measure the angle of each action, it is recommended to take a comfortable step forward or as far forward as possible (Haff and Triplett, 2016). In addition to varying knee movement, several additional technique variations are frequently used, such as varying the step length, trunk position, and performing lunges with additional external loading (i.e., dumbbells or barbells) (Farrokhi et al., 2008; Jönhagen et al., 2009; Riemann et al., 2012). The results of these studies indicate that the biomechanical characteristics of the forward lunge exercise varied with these variations. Specifically, forward lunge with trunk extension significantly increased the net moment impulse at the knee, whereas using a flexed trunk position increased the net moment impulse at the ankle and hip extensors (Farrokhi et al., 2008). Escamilla et al. (2008) have examined the effects of direction (Escamilla, 2008), strides and step length in healthy subjects, and the resultant effects on patellofemoral compressive forces. But, a recent systematic review indicated that all squat exercises can cause tension overload in the knee, especially with knee flexion between 60° and 90° degrees (Pereira et al., 2022). The effectiveness of adjusting step length for alleviating knee stress is uncertain. Although the exercise “lunge without stride” in that study is actually a split squat exercise. However, he did not standardize the step length of each subject, despite the fact that leg length or height varies among individuals, and only used two fixed lengths. Moreover, according to a study about the effects of adding external resistance on kinematics and kinetics, the addition of external weight did not change the peak flexion angles of the hip, knee, and ankle attained during the forward lunge. And external loading prompted increases in hip and ankle contributions but had a minimal effect on the knee (Riemann et al., 2012). In summary, although Schütz et al. examined three short step lengths of the split squat, there is a lack of research on the kinematics and kinetics of the lower extremity during various step lengths, particularly those that are equal to or greater than the length of the leg.

No studies have been conducted to observe the kinematics and kinetics of the lower extremity among multiple different step lengths creating a void in the existing literature.

The strength of the lower extremity muscles, particularly those around the knee, and the ratios between different muscle strengths, play a crucial role in rehabilitation and strength training. Some studies have pointed out through EMG that the vastus medialis (VM) and vastus lateralis (VL) are two of the key muscles that control the frontal plane kinematics of the knee (Wünschel et al., 2011), which may also influence the activation of other muscles. And the imbalance between the VM and VL has been associated with an anterior cruciate ligament injury and patellofemoral pain syndrome (Fagan and Delahunt, 2008; Irish et al., 2010). However, knee stability is also dependent on hamstring function, such as the activity of the biceps femoris (BF) (Danneskiold-Samsøe et al., 2009; Abulhasan and Grey, 2017). Imbalances in knee muscle strength can negatively affect strength training by causing pain, stiffness, and injuries during physical activity (Ibis et al., 2018).

The activation of specific muscles can be varied by performing variations on the same exercise. To our knowledge, performing variations of split squats or lunges may alter the muscle activation of the lower extremities, possibly resulting in changes in strength that may be important both for the rehabilitation of patients and the strength training of healthy adults. For example, certain types of squats and lunges result in different activation of the VM compared to the VL (Irish et al., 2010; Felicio et al., 2011; Jang et al., 2013). Specifically, split squats performed with greater hip adduction not only activate the VM more than the VL but also the gluteus maximus (GM) more than conventional squats (Felicio et al., 2011; Severin et al., 2017; Williams et al., 2021). In addition, the trunk and upper extremity position during the lunge exercise can significantly affect the muscle activation of the lower extremities (Farrokhi et al., 2008). In general, a forward lunge with the forward leaning of the trunk shows greater muscular action of the hip extensors when compared to the normal lunge with the trunk erect. In contrast, performing a forward lunge with the trunk extended did not alter the EMG signals of the lower extremity musculature. Therefore, it is important to choose the most appropriate step length to activate the right muscles. However, it is unclear how alterations in the step length of split squat affect the activations of the lower extremity muscles.

In summary, the objective of this study was to describe the kinematics, kinetics, and muscle activation of the lower extremities during split squat among four different step lengths. It was hypothesized that the peak angle and the extension moment of the hip increases with the increase of step length, and decrease for the knee. The EMG activity of the hip extensor increases with the increase of step length, and decrease for the knee extensors. The results of this study may provide theoretical support for guiding the selection of exercises or training programs, both for patients’ rehabilitation and healthy adults’ strength training.

## 2 Materials and methods

### 2.1 Subjects

Twenty male college students majoring in physical education who engage in regular exercise (at least twice a week) participated in this study (mean ± SD age, 23.9 ± 3.7 years). Participants who had previous neurological disease, hypertension, or orthopedic pathology were not included in this study. The mean height and mass of the participants were 1.75 ± 6.4 m and 81.2 ± 3.8 kg, respectively. The mean 1RM of the split squat was 1.1 ± 0.3 kg/BW. All participants were familiar with the split squat. The protocol for this study was approved by the Health Sciences Institutional Review Board (NO.2020187H), and participants provided their informed written consent prior to participation.

### 2.2 Instrumentation

Thirteen retroreflective markers, each 14 mm in diameter, were affixed to palpable body landmarks to estimate the rotational centers of the ankle, knee, and hip. Markers were placed bilaterally at the anterior superior iliac spine (ASIS), the top of the crista iliaca, the L4-L5 interface, the anterior thigh, the lateral and medial femur condyles, the lateral and medial malleolus, the tibial tuberosity, the center of the second and third metatarsals, and the heel of dominant limb. Three-dimensional kinematic data were collected using an 8-camera motion analysis system at 200 Hz (Motion Analysis Raptor-4, USA). Kinetic data were collected using force plates (Kistler Instrumente AG, Winterthur, Switzerland), which were embedded in the floor and sampled at 1000 Hz. The coordinate and ground reaction force signals were time-synchronized using Cortex, version 2.6.2 (Motion Analysis Corporation, Santa Rosa, United States).

Surface electromyography (EMG) of eight muscles, was recorded using silver-contact wireless bipolar bar electrodes with fixed 1 cm interelectrode spacing (Trigno, Delsys Inc., Natick, MA, United States). Electrodes were placed parallel to muscle fibers of gluteus maximus (GM), vastus lateralis (VL), vastus medialis (VM), rectus femoris (RF), biceps femoris (BF), Semitendinosus (ST), medialis gastrocnemius (MG), lateralis gastrocnemius (LG). A maximal voluntary isometric contraction (MVIC) was performed for each of the eight muscles to elicit maximal activity, as previously described in the literature (Anderson and Barnum, 2021), and to provide EMG normalization criteria. Prior to electrode placement, sites were shaved and skin scrubbed with isopropyl alcohol, and each of the MVIC tests was repeated twice.

### 2.3 Experimental design

At the first visit, the 10RM barbell weight for each subject’s split squat was determined by a standardized protocol. After a standardized warm-up (jogging and dynamic stretching), an estimated 10RM weight was selected for the split squat. When the maximum number of reps was greater than 10, the weight was increased until the maximum number of squats was 10. Oppositely, when the maximum number of reps is less than 10, decrease the weight until the maximum number of squats is 10. Each increase or decrease in weight is 10% of the estimated 10RM weight. During all the attempts, each participant was asked to squat until reaching a depth where the thighs were parallel to the floor. In addition, each subject’s 10RM barbell weights were determined using a comfortable step length.

During the second visit, all subjects were required to complete split squats under four step length conditions (50%, 70%, 100%, and 120% of leg length) while kinematic, ground reaction force, and EMG data were collected. During each condition, subjects were required to perform split squat exercises for three consecutive repetitions, using their 10-repetition maximum (10-RM) barbell weight. To control for multiple exposure and fatigue effects, each participant was randomly assigned a step length condition order. The data was collected mainly from the dominant lower extremity, which was positioned in front, while the non-dominant side was positioned behind. The dominant lower extremity was operationally defined as the preferred limb for kicking a ball. A 72-h resting period was given to participants between the 10RM procedure and the formal data collection (Sun and Yang, 2023).

### 2.4 Split squat procedures

**FIGURE 1:**
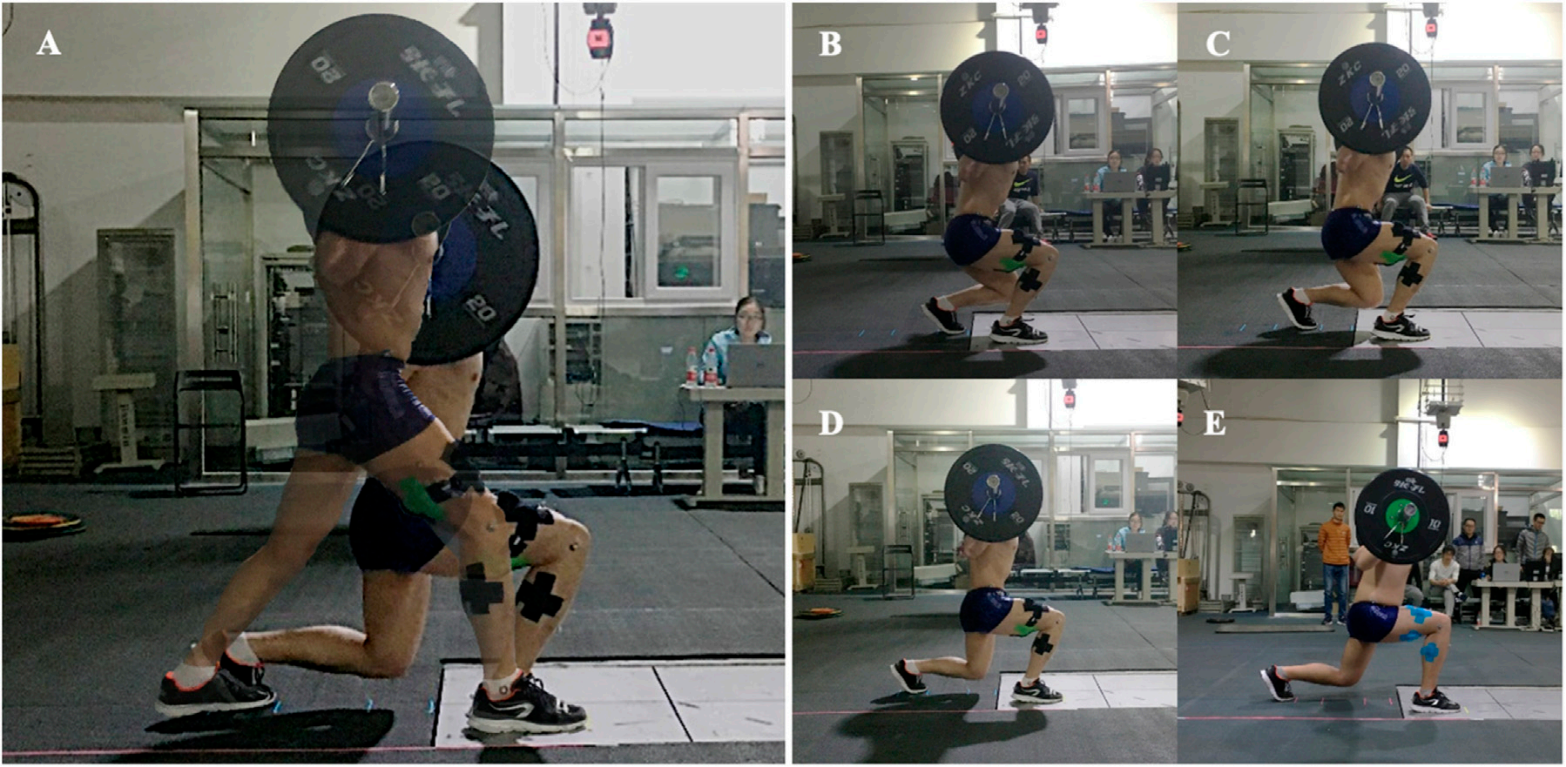
Split squat exercise and its variations for all four step length conditions: **(A)** Starting and ending postures; **(B)** 50%LL step length; **(C)** 70%LL step length; **(D)** 100%LL step length; **(E)** 120%LL step length.

Each subject participated in the preliminary experiment 72 h before the formal experiment. For the split squat exercise, all participants wore the same brand (Decathlon, France) and style of shoes. Four step lengths were determined for each subject based on their own leg length, and each subject was given the opportunity to practice the split squat for the four conditions. The stepping length for the split squat was standardized by using the leg length, which was measured from the greater trochanter to the lateral malleolus, and four different lengths were set at 50%, 70%, 100%, and 120% of leg length. Tape strips were placed on the floor at the starting point and target step length (Figure 1).

First of all, participants were instructed to step forward into a split stance with the dominant limb on the force plate and then complete each repetition by lowering the body until the front thigh was parallel to the floor. Once they reached the lowest position, they were instructed to immediately rise upward and return to the split standing starting position. They were also instructed to maintain an erect torso during the entire split squat. A video camera recorded a view of the sagittal plane from the right side, which was chosen to make sure the erect torso and thighs are parallel to the floor. Participants attempted to complete the lowering phase of each repetition within 2 s; an acoustic metronome set to 60 beats per minute was used to assist with movement. Several familiarization trials (3–6 trials) were allowed for each step length condition before data collection so that the participants could become comfortable with the movements and length.

### 2.5 Data analysis

Lower extremity kinematics, kinetics, and EMG data were collected simultaneously in all four step length conditions. The original 3-dimensional coordinate data of the markers were filtered using a Butterworth low-pass digital filter with an estimated optimal cutoff frequency of 13 Hz (Yu B et al., 1999). Visual 3D software (C-Motion, Rockville, MD) was used to quantify the motion of the hip, knee, and ankle based on the relative motion between adjacent segments. For example, the angles of the knee joint were determined by using Cardan angles of the shank reference frame in relation to the thigh reference frame, which were rotated in a particular sequence of flexion-extension, valgus-varus, and internal rotation-external rotation (Wu G et al., 2002). Joint extension moment were calculated using an inverse dynamic approach, and normalized to body weight (Greenwood D.T., 1988). The EMG signals were differentially amplified (CMRR >80 dB, input impedance 1,015 Ω), band-pass filtered (10–800 Hz), sampled at 2000 Hz, and converted with a 16-bit card with a ±5 V range. The average of the amplitude was calculated using the root mean square (RMS) method and were normalized according to maximal voluntary contraction (MVC). For each dependent variable, the average across the 3 trials within each of the 4 step length conditions was calculated and used for statistical analysis.

For each dependent variable, the average across the three trials within each of the four step length conditions was calculated and used for statistical analysis. One-way repeated measures analyses of variance (ANOVAs) were used to compare the split squat characteristic variables (peak angle of hip, knee and ankle; peak moment of hip, knee and ankle; and eight muscles’ RMS of the lower extremity) among the four step length conditions. In all analyses, a Greenhouse-Geisser correction factor was applied when sphericity was indicated. The statistical significance was set at *p* < 0.05. When statistical significance was evident for the four split squat characteristics, LSD *post hoc* tests were used. All statistical tests were conducted in Statistical Package for Social Science software (SPSS, version 22.0, United States).

## 3 Result

### 3.1 Peak flexion (ankle dorsiflexion) angles

**FIGURE 2:**
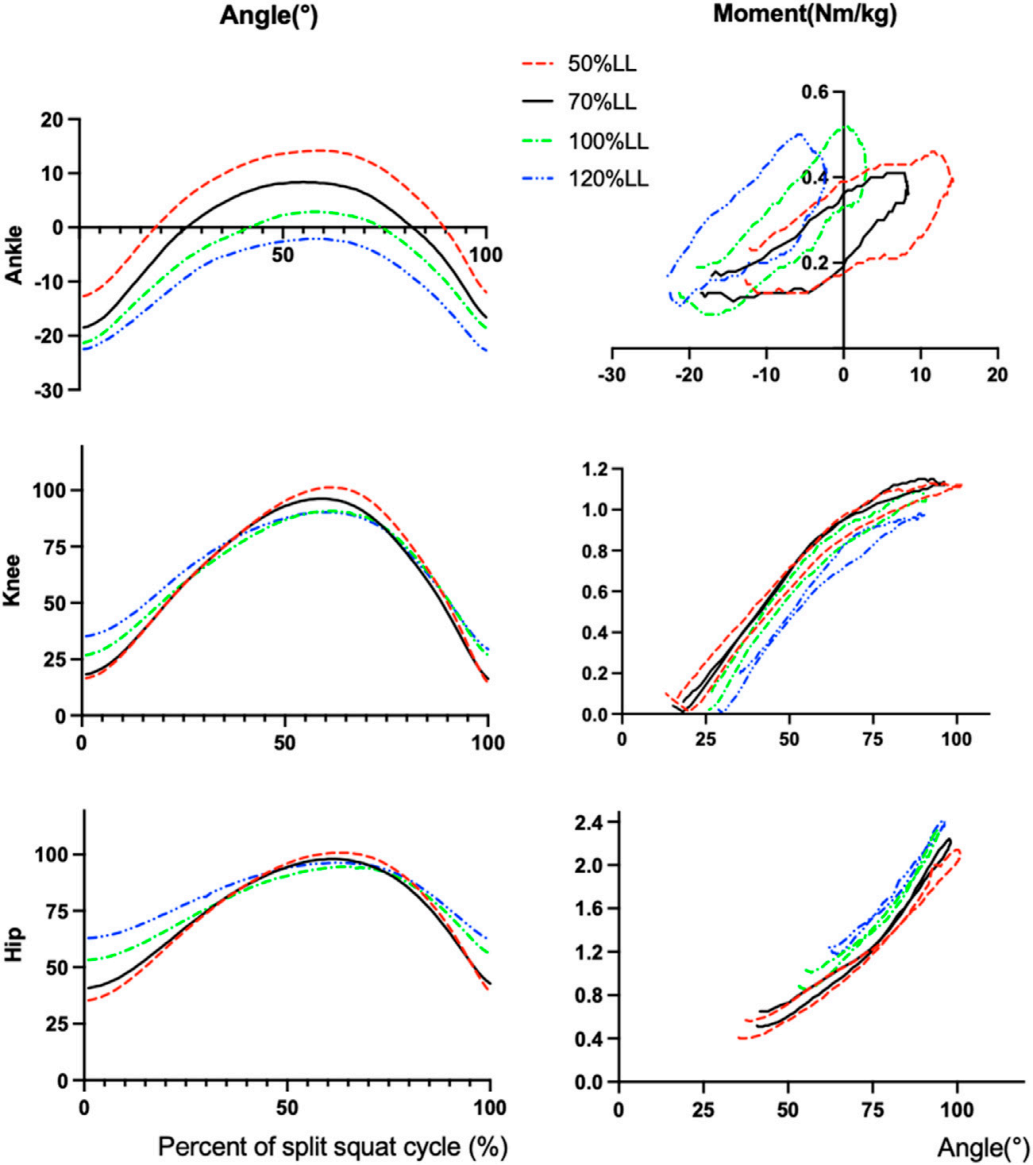
The angle and extension moment for hip, knee and ankle across the 4 step length conditions during the split squat. Abbreviations: 50%LL, 50% of leg length; 70%LL, 70% of leg length; 100%LL, 100% of leg length; 120%LL, 120% of leg length.

Descriptive statistics for the peak flexion angles are presented in Table 1; Figure 2. The results of variance analysis showed that there were significant differences in the peak flexion angles of the hip joint (*p* = 0.011), knee joint (*p* < 0.001), and ankle joint (*p* < 0.001) among different step lengths. Post hoc analysis revealed that the peak hip flexion angle at 100% LL step length was significantly smaller than that at 70% LL step length (*p* = 0.005), and 120% LL step length (*p* = 0.019). The flexion angle at 50% LL step length tended to be greater than that at 100% LL step length (*p* = 0.064). The peak knee flexion angle at 100% LL step length was significantly smaller than that at 50% LL (*p* = 0.002) and 70% LL (*p* < 0.001) step length. The angle at 120% LL step length was significantly smaller than the angle at 50% LL (*p* < 0.001) and 70% LL (*p* = 0.001) step length. The peak ankle flexion angle at 120% LL step length was significantly greater than the angle at 50% LL (*p* < 0.001), 70% LL (*p* < 0.001), and 100% LL (*p* < 0.001) step length. The peak ankle flexion angle at 100% LL step length was significantly greater than 50% LL (*p* < 0.001), and 70% (*p* < 0.001) step length. The peak ankle flexion angle at 70% LL step length was significantly greater than the angle at 50% LL (*p* = 0.048) step length.

**TABLE 1:** Peak flexion angles for the hip, knee, and ankle across the 4 step length conditions during the split squat (Mean ± SD).

	Step length condition, peak flexion Angle,°					
Joint^#^	50% LL (a)	70% LL (b)	100% LL (c)	120% LL (d)	*p*	Post-hoc
Hip	101.1 ± 9.9	101.9 ± 10.0	97.9 ± 8.2	100.5 ± 7.8	0.011*	c < b, c > d
Knee	104.0 ± 9.3	102.1 ± 7.9	96.3 ± 7.2	95.4 ± 8.7	<0.001*	c < a/b, d < a/b
Ankle	−14.2 ± 5.8	−8.4 ± 5.3	−2.9 ± 4.6	2.1 ± 6.8	<0.001*	b < a, c < a/b, d < a/b/c

Abbreviations: 50% LL, means 50% leg length; 70% LL, 100% LL, 120% LL, means 70%,100%, 120% leg length respectively.

Negative Positive values indicate ankle dorsiflexion.

^#^Joint main effect: hip, knee, ankle.

*p < 0.05.

### 3.2 Peak net joint moment

Descriptive statistics for the peak net joint moment are presented in Table 2; Figure 2. Positive value indicates extension moment. The results of variance analysis showed that there were significant differences in peak extension moment of hip joints (*p* < 0.001) and knee joints (*p* = 0.002) among different step lengths, and no significant differences in ankle peak extension moment (*p* = 0.357). Post hoc analysis revealed that the peak extension moment of the hip joint at 120% LL step length was significantly greater than that at 100% LL (*p* = 0.013), 70% LL (*p* = 0.008), and 50% LL (*p* = 0.008) step length. The peak hip extension moment at 50% LL step length was significantly smaller than that at 70% LL (*p* = 0.008) and 100% LL (*p* < 0.013) step length.

**TABLE 2:** Peak net joint moment for the hip, knee and ankle across the 4 step length conditions during the split squat (Mean ± SD).

	Step length condition, peak net joint moment, N·m/kg					
Joint^#^	50% LL (a)	70% LL (b)	100% LL (c)	120% LL (d)	*p*	Post-hoc
Hip	1.56 ± 0.22	1.66 ± 0.21	1.72 ± 0.37	1.80 ± 0.29	<0.001*	b < a, c < a, d < a/b/c
Knee	0.86 ± 0.15	0.89 ± 0.13	0.84 ± 0.15	0.77 ± 0.17	0.002*	b < a, c < a/b, d < a/b/c
Ankle	0.42 ± 0.07	0.41 ± 0.10	0.44 ± 0.14	0.43 ± 0.13	0.357	—

Positive value indicates extension moment.

^#^Joint main effect: hip, knee, ankle.

*p < 0.05.

### 3.3 EMG root mean square

Descriptive statistics for muscle activation are presented in Table 3. A significant difference was observed in the muscle activity of the GM muscle (*p* < 0.001), the VM (*p* = 0.013), the VL (*p* = 0.020), the BF (*p* = 0.003), the ST (*p* < 0.001), the MG (*p* = 0.035) and the LG (*p* = 0.005) during the lunge for all step length, and no significant difference in RF (*p* = 0.16). In the *post hoc* test. The root mean square amplitude (RMS) for GM at 50% LL step length was significantly smaller than the RMS at 70% LL (*p* = 0.020), 100% LL (*p* < 0.001), and 120% LL (*p* < 0.001) step length, and 70% LL step length was significantly smaller than the RMS at 120% LL step length (*p* = 0.037). The RMS for VM at 50% LL step length was significantly smaller than the RMS at 100% LL (*p* = 0.010) and 120% LL (*p* < 0.012) step length. The RMS for VL at 50% LL step length was significantly smaller than the RMS at 70% LL (*p* = 0.006) step length, and 120% LL step length was significantly smaller than the RMS at 100% LL step length (*p* < 0.001). The RMS for BF at 50% LL step length was significantly smaller than the RMS at 70% LL (*p* = 0.038), 100% LL (*p* < 0.001), and 120% LL (*p* < 0.001) step length, and 70% LL step length was significantly smaller than the RMS at 120% LL step length (*p* < 0.001). The RMS for ST at 50% LL step length was significantly smaller than the RMS at 70% LL (*p* = 0.045), 100% LL (*p* = 0.002), and 120% LL (*p* < 0.001) step length, and 70% LL, 100% LL step length was significantly smaller than the RMS at 120% LL step length (*p* < 0.001). The root mean square amplitude (RMS) for MG at 50% LL and 70% LL step length was significantly smaller than the RMS at 100% LL (*p* = 0.004, *p* = 0.048) and 120% LL (*p* = 0.048, *p* = 0.015) step length, respectively. The RMS for LG at 50% LL and 70% LL step length was significantly smaller than the RMS at 100% LL (*p* = 0.003, *p* = 0.001) and 120% LL (*p* = 0.012, *p* = 0.002) step length, respectively.

**TABLE 3:** Muscle activation across the 4 step length conditions during the split squat (Mean ± SD).

	Step length condition, %MVIC					
Muscle	50%LL	70%LL	100%LL	120%LL	*p*	Post-hoc
GM	0.87 ± 0.67	1.14 ± 0.91	1.27 ± 0.88	1.30 ± 0.85	<0.001*	b < a, c < a, d < a/b
VM	1.24 ± 0.55	1.28 ± 0.46	1.40 ± 0.75	1.42 ± 0.82	0.013*	c < a, d < a
RF	1.12 ± 0.77	1.17 ± 0.61	1.11 ± 0.54	1.09 ± 0.45	0.16	—
VL	0.80 ± 0.52	1.04 ± 0.68	0.91 ± 0.54	0.79 ± 0.48	0.020*	b < a, d < c
BF	0.58 ± 0.52	0.66 ± 0.53	0.72 ± 0.69	0.78 ± 0.78	0.003*	b < a, c < a, d < a/b
ST	0.28 ± 0.19	0.33 ± 0.23	0.36 ± 0.24	0.45 ± 0.26	<0.001*	b < a, c < a, d < a/b/c
MG	0.40 ± 0.51	0.32 ± 0.21	0.47 ± 0.54	0.53 ± 0.45	0.035*	c < a/b, d < a/b
LG	0.49 ± 0.46	0.45 ± 0.32	0.64 ± 0.52	0.74 ± 0.52	0.005*	c < a/b, d < a/b

Abbreviations: MVIC, maximal voluntary isometric contraction; GM, gluteus maximus; VM, vastus medialis; RF, rectus femoris; VL, vastus lateralis; BF, biceps femoris; ST, semitendinosus; MG, medialis gastrocnemius; LG, lateralis gastrocnemius.

*p < 0.05.

## 4 Discussion

The purpose of this study was to compare the differences in kinematic, kinetic, and muscle activation characteristics of the anterior lower extremity when performing a split squat with leg length-based standardized step lengths in healthy adults. There is a significant change in the peak flexion angle of the hip joint. The range of motion (ROM) decreased, while the peak extension moment increased with the increase in step length. Peak knee flexion angle, knee extension moment, and peak ankle angle decrease with increasing step length. In general, a strength exercise is executed safely within the physiological ROM of a joint and by avoiding overloading of the human tissue (Kirkby Shaw et al., 2020). Our results provide a theoretical basis for patients or healthy adults to make choices when training with specific split squat technique variations.

### 4.1 Hip kinematics and kinetics

**FIGURE 3:**
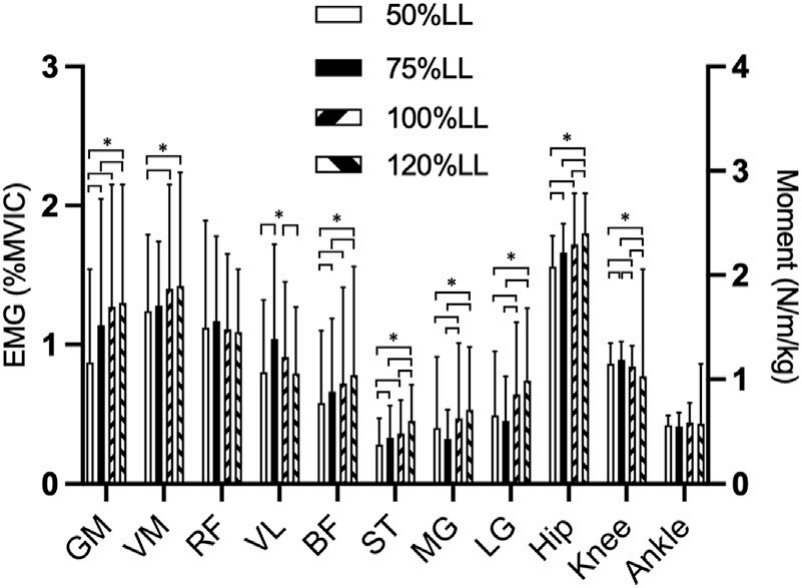
Comparison of each muscles EMG and each joint moments during 4 step length conditions of the split squat Abbreviations: 50%LL, 50% of leg length; 70%LL, 70% of leg length; 100%LL, 100% of leg length; 120%LL, 120% of leg length. GM, gluteus maximus; VM, vastus medialis; RF, Rectus femoris; VL, vastus lateralis; BF, biceps femoris; ST, Semitendinosus; MG, medialis gastrocnemius; LG lateralis gastrocnemius. *p < 0.05.

In order to avoid the effects of trunk angle and squat depth on the biomechanical characteristics of the lower extremity among four step lengths, we asked all subjects to keep the torso upright and squat down to the position where the front thigh was parallel to the ground at four split lengths. Therefore, in theory, step length will not affect the peak flexion angle of the hip joint, but the experimental results are inconsistent with our hypothesis. The peak flexion angle of the hip at 100% LL step length was significantly smaller than at 70% and 120% LL step length (Figure 3). The reason for this analysis may be that our subjects were more accustomed to performing lunge squats at 100% LL step length in their normal practice and habitually reached the squat depth they normally train at, rather than strictly reaching the position required in our experiment.

As shown in our data, the ROM of the hip joint gradually decreases and the peak net moment gradually increases with increasing step length, which is more challenging for the hip extensors. Our results supported the work of Riemann et al. (2013) which also observed that the long step length of lunge required more net hip extension moment. However, there was no significant difference in the peak hip extension moment between 70% LL and 100% LL of the step length, which was hypothesized to be possibly related to the insufficient squat depth of the subjects at 100% LL, as evidenced by the kinematic data of the hip joint.

As S. P. Flanagan et al. (2004) show the split squat is a hip extensor-dominant exercise. During his exercise in older adults, the hip joint exhibited higher peak net joint moment, net joint moment impulse (NJMI), and mechanical energy expenditure compared to the knee and ankle joints, providing 53% of the total support impulse while the knee and ankle provided 26% and 21%, respectively (Flanagan et al., 2004). During the four conditions in our study, the peak extension moment of the hip is much greater than that of the knee and ankle. Although we use the peak joint extension moment instead of the extension moment impulse, all support greater contributions to the split squat by the hip than either the knee or ankle. Interestingly, the results of this experimental study showed a 15.7% increase in peak hip extension moment and a 15.1% decrease in the knee when increasing the step length (50% LL to 120% LL). Probably, increasing the distance between the feet in a split squat can shift the load from the knee to the hip joint, which can be beneficial for patients with knee injuries who need to avoid placing too much stress on the knee joint during rehabilitation.

### 4.2 Knee kinematics and kinetics

The split squat is a movement dominated by the front lower extremity (Hofmann et al., 2017). Theoretically, the greater the step length, the smaller the maximum knee flexion angle of the front lower extremity when the relative position of the center of gravity between the legs does not shift back and forward. Our findings confirm this trend, although the maximum flexion angle of the knee was only significantly different between the larger step (>100% LL) and smaller step (<70% LL), while there was no significant difference between 100% LL and 120% LL, and between 70% LL and 50% LL step lengths (Figure 3). We speculate that although not every increase in step length causes a significant change in peak knee flexion angle, there are areas between 70% LL and 100% LL step length that are “sensitive zone” to changes, i.e., changes in step length can cause a correspondingly significant change in peak knee flexion angle. We found that performing split squats at a step length away from this “sensitive zone” produced smaller changes in knee angle. For rehabilitation patients, a stabilized movement with limited variation is very meaningful, especially for rehabilitation patients with limited knee motion. This result is recommended for those beginners with unstable movements to choose ≥100% LL step length for split squat exercises.

The peak knee extension moment showed a gradual overall decrease with increasing step length but was significantly greater at 70% LL step length than at 50%. The reason could be the over-short step length (50% LL) during split squatting causing a backward shift in the body’s center of gravity, leading to increased load on the posterior limb. But our experiments did not collect data from the posterior limb, which is a limitation of our study. However, some studies (Schütz et al., 2014) showed that the weight shared by the posterior limb increases as the step length or tibial angle changes, which also verifies our conjecture from the side. It is hoped that subsequent research around changes in step length and anterior-posterior lateral limb forces can be continued.

Many previous studies have shown that excessive peak extension moment in sports may cause cartilage or ligament tissue damage (Myer et al., 2015; Thompson et al., 2017), and Patellofemoral Joint Stress is also positively correlated with peak knee extension moment (Boling et al., 2009), therefore excessively small split squat step lengths are not advocated for knee rehabilitation (e.g., post-ACL rehabilitation, patients with patellar stress syndrome).

### 4.3 Ankle kinematics and kinetics

Many adults and even athletes will have varying degrees of dorsiflexion restriction, which is an important factor in athletic performance or injury (Hamstra-Wright et al., 2021). The maximum ankle dorsiflexion angle in the split squat decreases with increasing step length, with a maximum foot dorsiflexion angle of 14.2° ± 5.8° at 50% LL step length, which is a challenge for athletes with limited foot dorsiflexion or for rehabilitation after a foot and ankle surgery (Comfort et al., 2015).

At 120% LL step length, the split squat is entirely in the plantar flexion of the foot, which means that the entire process of squatting works out the calves within the vertical line. Therefore, we recommend that for some patients with limited dorsiflexion of the foot, split squat training be performed with ≥100% LL of the step length. Despite greater variation in peak ankle dorsiflexion angles at different step lengths, there was no statistically significant difference in peak ankle extension moment.

Our results seem to indicate that increasing step length can lead to more favorable kinematics and dynamics of the lower extremity joints. However, during the practice process, it became evident that excessively large step lengths (e.g., ≥120%LL) negatively impact movement maneuverability, often resulting in forward slipping. Notably, significant improvements were not observed beyond a step length of 100%LL.

### 4.4 Lower extremity muscle EMG analysis

The kinematic and kinetic data for a given movement reflect the outward appearance of all muscles involved in the movement, EMG provide a good indication of the activation of a muscle during a particular phase of a given movement. The results of the EMG data in this study can reflect the activation of the relevant muscles during the split squat movement at four step lengths (Figure 3). The split squat has been identified as a hip-dominant movement in numerous studies, regardless of the knee and ankle joints’ involvement (Farrokhi et al., 2008; Schütz et al., 2014). Our kinetic and EMG data also validate this conclusion. In summary, our findings indicate that the alterations in step length have more activation on the muscles located in the posterior pelvis (GM), posterior thigh (BF, ST) and shank (MG, LG) while having a relatively minor effect on the muscles of anterior thigh (VM, LM, RF).

There are many clinicians and health professionals who recommend their patients strengthen the quadriceps with split squat or lunge movements. Therefore, appropriate training movements are important to optimize muscle activation. Same as our hypothesis, the activation of the hip extensors, knee extensors, and flexors was significantly less at 50% LL step length than the other three lengths. We can speculate that this may be due to the small step length of the split squat, thus increasing the load on the posterior lower extremity and reducing the activation of the anterior lower extremity muscles. Hofmann’s study also demonstrated that reducing step length resulted in more weight-bearing on the posterior limb (Hofmann et al., 2017).

Anatomically, the RF serves the dual function of flexing the hip and extending the knee, while the BF and ST perform the opposite functions. Therefore, it can be challenging to determine whether the RF and BF/ST is engaging in a centrifugal or centripetal contraction during a split squat (Henriksen et al., 2009). However, research has suggested that the BF and ST have greater moment arms at the hip joint than at the knee joint (Jönhagen et al., 2009). As the step length increases, the moment arm of the BF and ST at the knee joint increases. There was no significant difference in the EMG of the RF at the four steps which is similar to the results of a previous study (Bezerra et al., 2021). Both are also polyarticular muscles of the thigh, however, the EMG of the BF and ST muscles increased with an increase in step length. The reason could be that the BF and ST muscles experience a greater changes in moment arm at the knee joint compared to the RF muscle.

Consistent with the previous results (Pincivero et al., 2004), the EMG of VM was greater than the VL and RF muscles across all four step lengths. In our study, the knee joint angle was almost 90° at the moment of peak extension moment, with a 120% LL step length. These findings were consistent with the results of Pincivero’s (Pincivero et al., 2004) study which showed a greater improvement in recruitment efficiency for the VM muscle as compared to the VL and RF muscles, specifically from 70° to 90° flexion. Although the EMG of the VM varied in step variations but was only limited to the maximum step length (120% LL) showing a stronger EMG. It is speculated that the difference demonstrated in VM may be due to the excessive weight and the large step length used in our study. Additionally, taking steps greater than 100% LL can jeopardize the stability of the training movement, ultimately leading to limited gains. The opposite is true for VL, where EMG is greater at smaller steps (75% LL).

The EMG of MG and LG was only significantly different between the longer (100% LL and 120%LL) and shorter (50% LL and 70% LL) step length. We hypothesized that a 10RM load would result in a higher demand for ankle stability when the step lengths are larger. These all results may provide a clearer training protocol for patients rehabilitating from clinical muscle atrophy but also requires consideration of whether the patient’s knee joint mobility and maximum net joint moment are within the patient’s tolerance range.

## 5 Conclusion

With the step length variations, the ROM of the hip, knee, and ankle joints, peak joint net moments, and the EMG of lower extremities underwent changes. Specifically, with the step length increases, the ROM in the knee and ankle joints tends to decrease, while the peak extension moment of the hip joint increases. In addition, alterations in step length had a greater influence on the hip extensor muscles, while having limited impact on the knee extensor muscles, especially between 100% LL and 120%LL. But in practice, if the length of the step goes beyond 120% LL of the step length, it would require more stability and induce only minor enhancements in muscle activation. It is recommended that the appropriate step length should be selected based on specific needs, such as considering joint restrictions, avoiding excessive joint stress, or increasing specific muscle activation when customizing rehabilitative exercise prescriptions. The optimal step length for strength training in healthy adults appears to be more suitable when it is equal to the length of the individual lower extremity.

## Data Availability

The raw data supporting the conclusion of this article will be made available by the authors, without undue reservation.
